# Parliament and the Professions

**Published:** 1920-02-28

**Authors:** 


### PARLIAMENT AND THE PROFESSIONS.

### Military Mental Hospitals

In reply to a question as to alleged ill-treatment of patients in Army mental hospitals, Mr. Churchill said that strict orders were issued by the Director-General, Army Medical Service, that every complaint, however made and of whatever nature, should be immediately inquired into. He was satisfied that the general allegations made against the officials of the Army mental hospitals and the Army Medical Service are entirely unjustified.

### Army Medical History Sheets.

Colonel Ashley asked the Secretary of State for War whether he will consider the question of furnishing each man on discharge or demobilisation with a copy of his Army medical history sheet, as the possession of this evidence would very materially assist men in establishing their statutory right to a pension. Sir A. Williamson answered to the effect that the history sheets are confidential, and it would be contrary to the practice of the War Office to furnish copies of them to the men concerned. Apart from this, a great deal of additional clerical work would be involved. The medical history of invalided soldiers are sent to the Ministry of Pensions, who are able to get any others required from the Record Office.

### Municipal Maternity Homes.

Dr. Addison stated that the Local Government Board and the Ministry of Health have for some time pressed upon local authorities, both in general circulars and in individual letters, the importance of the provision and maintenance of maternity homes, and have obtained Treasury sanction for a grant of half the approved expenditure on these purposes. A memorandum has just been issued, and will be placed on sale, giving guidance as to suitable plans and equipment and so forth. During the last year or so about twenty-five such homes were established by municipal authorities, and about twenty by voluntary bodies, who, as a rule, received financial assistance from local authorities. Many similar homes have been planned.

